# Notices

**Published:** 1869-08

**Authors:** 


### NOTICES.

Five Acres too Much, by Robert B. Roosevelt, author of "Game Fish of North America," "Superior Fishing," "Game Birds," etc. Published by Harper Brothers in a very attractive, cheery garb, is so irrisistibly amusing, and, withal, so common-sensical, that it merits the perusal of every gentleman farmer, every Amateur--Horti—Flori—Agri—Fructi—Berri —Piggi—Henni—and every other kind of "culturist" ever heard of, being a truthful elucidation of country attractions; it is brimful of fun, and will do an invalid more good than fourteen pints of Peruvian Syrup, or sixteen hundred Brandreth Pills. Whoever believes in the sanitary effects of good laughs "encored" *ad infinitum*, should give a dollar and a half for the book.

Mother at Home, edited by Mrs. Henry Ward Beecher, 32 Cedar street, New York, $1.50 a year, published monthly, treats of many things connected with the family in the July number. Every young housekeeper should take it.

The Little Sower Monthly. $1.25 a year. Indianapolis, Ind. Is profusely illustrated, and furnishes a large amount of instruction and amusement for a family.

The Mother's Journal is promptly on our table, and it comes looking as beautiful as May itself. This illustrated

Magazine for the home is one of the best in the circle of Magazines. The gem of a picture we cannot give you—the contents are as follows: "Co-operative Housekeeping," "Spring and Sweet May," "Letters to Young Mothers," "I didn't see it," "Work a Blessing," "The Mother's Vision," "The Widower and the Motherless," "Julia Waldron's First Term," "Let the Children be Consecrated," "Joan of Arc," "Management of Family Affairs," "Letter from London," "Crazy Kate," "Dressing for Church," "Mamma's Asleep," "Nelly Melrose," "Familiar Chat upon Fashions," "Literary Notices," Chicago: Price $2.

Papers Over the Water, by Sinclair Tousey. 204 pp., 12mo. Issued by the American News Co., New York. This is the most concise and instructive book of travel which we have seen for a long time. The writer enters a village or town or city, sees at a glance what is distinctive, puts it down in a few telling Anglo-Saxon words, then hies away to some other point. For terseness, fidelity, comprehensiveness, and for quiet, suggestive humor on every page, it has no superior in that line of books.

Knee-Sprung Horses permanently cured. Receipt one dollar. Address W. T. Baker, *Sentinel* office, Waterford, New York.

The Sanitary Brace, patentee G. F. Pinckard, New Orleans, La., is intended to teach people how to keep their mouths shut. A man once said, "Lincoln ought to be hung." Three years later it caused his bankruptcy and a violent death. The machine also prevents snorting and snoring; it also tends to prevent consumption and empty pockets, for it promotes health and thus gives power to work and make money. We advise all blabbers, snorters and snorers to send Mr. Pinckard ten cents for a pamphlet. It will pay.

Saratoga Springs.—The Albion House, new and convenient, with all modern improvements, adjoining the beautiful residence and gardens of Judge Crane, under the management of J. Howland, Esq., P. O. box 400, will be found a desirable place to all who wish quiet and comfort at a moderate price.

Zero Refrigerator.—Hundreds of our best citizens, and thousands of families throughout the country consider this indispensable family convenience the coolest, cleanest and sweetest refrigerator yet devised, hence it is rapidly superseding all others. It is so contrived that, while it keeps provisions of every description uniformally cool in summer, it prevents them from freezing in winter. It does not acquire a closet smell because it is lined with zinc and all the shelving is metallic. The ice is put in at the top and the provisions through an opening in front, so that the two never come in contact. Dampness is impossible where the provisions are, hence they are always kept sweet.

The ice being uppermost and the articles to be preserved under it and at either side, the air is always cool and dry. The ice is kept in a close box, and not being exposed to a circulation of air, a small amount keeps a low temperature; besides, the arrangement is such that cool drinking water is always at hand. The whole is filled with powdered charcoal, which is essential to the cleanliness of this ornamental piece of furniture, which has repeatedly taken the highest premium at various State fairs.

Gothic Furnace for burning coal and wood is economical, powerful and durable. The prices range from $100 to $150. Numerous testimonials have been forwarded from various States as to the superiority of the Furnace, and as many persons are building houses into which they hope to move in the winter, it would be well to call and see Mr. Leslie's stock before purchasing elsewhere, at 1310 Broadway, New York, or 605 Sixth Avenue.

Dangerous medication. Beneficial tendencies of Young Men's Christian Associations. Sacrifice of lives. Bad Air.

FEBRUARY. Essence of Disease. The nature of sickness. Philosophy of all remedial means. Selfishness. How families are to grow up lovely and loving one another. Washing the face and hands. Tight boots, effects, prevention and remedy. Breathing. Cold feet. Best slippers. Health and Thrift. Ash sifters. Unhealthy houses. Sleeping habits. Fire on the hearth. Best method of heating the family sitting room.

MARCH. The healthful influence of Bible principles in the conduct of life. Multiply and replenish the earth; the wickedness of thwarting the natural tendencies, &c. &c. A common cold; its prevention and cure. Wakefulness and how to prevent it. Common sickness. Where to live cheaply abroad: Dresden; Heidelberg; cost of living, schooling, and clothing. Sea voyages. To determine buggy sugar; loaf sugar only pure and clean. Soaps suds; "settling" water. Poisonous moulds. Bed bug riddance. The early decay among students and clergymen, and the cause.

APRIL. Hydrophobia. Phrensy. Pulpit and Pew. Responsibilities. Mothers' influence. May moving. Spring Diseases. Stewart's poor girls' hotel; rooms and board for two dollars a week, &c.

MAY. Physiology of preaching; the best preachers have the best health, as witness Beecher, Hall and Spurgeon; the characteristics of each; source of renown; the value of the voice and a distinct enunciation; have a clear idea, an earnest heart. Magnetic influence. Resignation. Marrying blood relations. Changing clothing. Walking erectly. Self reliance. How to reprove. Table manners, &c.

JUNE. Summer recreation; different kinds required by the different kinds of occupations and classes of society. The Adirondack Mountains. Going abroad. How much can be done with three month's time and five hundred paper dollars. Bronchitis, consumption and throat ail; the nature, causes and symptoms of each and how to distinguish them; the combination of symptoms which are curable, and those which are fatal; how to distinguish these diseases, &c.

JULY. Is the "eight hour" law a wise one? Woman's Rights; will voting add to her happiness? will it elevate or degrade? Dyspepsia. How to eat wisely. Cold feet. Constipation. Health's three essentials. Failing eyesight. Sleeping. How to avoid taking cold for a year, 15 cts. a number.

FOR LESS THAN NOTHING.

| | | | | | |
|---|---|---|---|---|---|
| Atlantic Monthly, . | . $4.00. | Journal of Health, . | . $1.50. | Both . | $3.90 |
| Galaxy, . . . . . | . 4.00. | " | " 1.50. | Both . | . 3.90. |
| Harper's Monthly, . | . 4.00. | " | " 1.50. | Both . | . 3.90. |
| Harper's Weekly, . | . 4.00. | " | " 1.50. | Both . | . 4.00. |
| Harper's Bazar, . | . 4.00. | " | " 1.50. | Both . | . 4.00. |
| Putnam's Magazine, | . 4.00. | " | " 1.50. | Both . | . 4.00. |
| London Lancet, . | . 5.00. | " | " 1.50. | Both . | . 5.00. |
| Phrenological Journal, | 3.00. | " | " 1.50. | Both . | . 3.00. |
| Rural New Yorker, | . 3.00. | " | " 1.50. | Both . | . 3.00. |
| Independent, . . . | . 2.50. | " | " 1.50. | Both . | . 3.00. |
| Riverside Magazine, | . 2.50. | " | " 1.50. | Both . | . 3.00. |

Any other four dollar magazine published in the United States, will be furnished for one year with Hall's Journal of Health for one year for three dollars and ninety cents; thus both publications may be had for one year for less than the price of the larger one.

Any three dollar publication in the United States, with Hall's Journal of Health, for 1869, will be furnished for $3.

If any of our present subscribers want any of the above or other publications now, they can have them on the same terms with The Journal of Health for 1870. Any of our one dollar and a-half books, or other books of same price will be sent, pp., with three copies of Hall's Journal of Health a year for $5.

### NOTICE.

In all cases let the money be sent in the form of a post-office order, payable to the order of W. W. Hall. Checks on banks or individuals for small amounts are sometimes troublesome of collection. A post-office order is perfectly safe for all parties; those who send money in letters must take the risk.

Although all the articles in Hall's Journal of Health are written by the editor himself, he feels justified in saying that in any number there is practical information as to the conduct of life in reference to health and disease which will be of more value to any one who will carry out the instructions, than fifty times the cost of a yearly subscription; there is no similar journal published in the world; its teachings are mainly derived from the editor's personal experience and observation, expressed in as few words as possible, and always plain and easily understood. Half of the ordinary diseases of life would be banished from society if the general instructions given in a few articles of the number for July, 1869, were observed.

KNEE SPRUNG HORSES permanently cured without cost or trouble. Address W. T. Baker, Sentinel Office, Waterford, N. Y.

